# Cytokine expression in gingival crevicular fluid around teeth opposing dental implants and 3-unit fixed partial dentures in a cross-sectional study

**DOI:** 10.1186/s13005-023-00359-0

**Published:** 2023-04-11

**Authors:** Xin-Rui Zhu, Chen-Xi Wang, Chang Chen, Rui-Yong Wang, Yu Zhang

**Affiliations:** 1 Department of Stomatology, Beijing Haidian Hospital, Beijing, 100080 China; 2 Department of Oral Implantology, Peking University School and Hospital of Stomatology, Beijing, 100081 China; 3 Department of General Dentistry, Shanghai Ninth People’s Hospital, College of Stomatology, Shanghai Jiao Tong University School of Medicine & National Clinical Research Center for Oral Diseases & Shanghai Key Laboratory of Stomatology & Shanghai Research Institute of Stomatology, Shanghai, 200011 China

**Keywords:** Gingival crevicular fluid, Cytokines, Dental implants, Fixed partial dentures, Contact teeth

## Abstract

**Objective:**

This study aimed to study the cytokines in gingival crevicular fluid (GCF) of the teeth opposing to dental implants and 3-unit fixed partial dentures (FPDs).

**Materials and methods:**

A total of 74 participants were recruited for this cross-sectional study. Based on the status of lower first molars, the participants were divided into dental implants group and 3-unit FPDs group. Social index and oral hygiene were recorded. Occlusal loading was evaluated with a T-scan. GCF was sampled from the upper first molar and assessed with a commercial cytokine assay kit.

**Results:**

Forty three dental implants patients and 31 3-unit FPDs patients received all of the clinical and laboratory evaluation. The dental implants group had a higher occlusion force distribution on first molars region. IL-10, IL-17, RANK had a higher mean in dental implants group and was associated with occlusion force of first molar. There was a weakly association between IL-10 and dental implants in the binary logistic regression analyses.

**Conclusions:**

In this study, the teeth opposing implants have a higher level of cytokines in the GCF than teeth opposing to 3-unit FPDs in periodontal healthy participants because of the poor osseoperception of dental implants. IL-10 might reflect a higher occlusion force in dental implants region.

**Clinical relevance:**

This study provided that different tooth restoration methods could influence the periodontal status of the contact teeth.

## Introduction

The first molar is very important for occlusal function, but its loss rate is high due to dental caries and other factors. Three-unit fixed partial dentures (FPDs) are a common restoration method for single missing teeth and have predictable clinical results. However, the preparation of abutments on both sides of the missing teeth space may cause sensitive pain and periodontal problems of teeth [1]. Dental implants have the advantages of minimizing damage to adjacent teeth and are currently widely used in clinical practice. However, there are many differences between implants and natural teeth as supporting units. One of them is that the lack of periodontal ligament further leads to a decrease in vascular distribution and neurotization between osseointegration implants and natural bone, which leads to poor bone perception and progressive occlusal overload [2]. Occlusal overload easily damages the periodontal tissue of natural teeth and leads to chronic inflammatory processes [3, 4]. Studies have shown that appropriate low occlusal force can exert anti-inflammatory, anti-catabolic, bone-protective and anabolic effects on periodontal tissue; in contrast, occlusal force overload can damage periodontal tissue, which in turn leads to a large number of cytokines being released from the damaged tissue, promoting the development of inflammation [5–7]. Various cytokines, such as interleukin (IL), interferon, and tumor necrosis factor (TNF), directly reflect the inflammatory state of periodontal tissue [8].

Gingival crevicular fluid (GCF) is the serum exudate of healthy individuals or inflammatory exudate composed of periodontal tissue cells, serum and oral bacteria, which contains cytokines. Considering that GCF can be collected noninvasively from periodontal pockets, more recent studies have focused on its cytokine expression under different conditions [9, 10]. In addition, compared with clinical parameters such as periodontal probing depth (PPD) and bleeding index (BI), cytokines are considered to be more specific in evaluating the site specificity of periodontal tissue [11]. In summary, detecting cytokines in GCF may be a favorable method to evaluate the periodontal state of teeth compared with dental implants and 3 units of FPDs [12]. At present, the research mainly focuses on the prosthesis and its influence on the surrounding tissues, and there are few studies to investigate whether 3 units of FPDs and implants will have different effects on the periodontal tissues of opposing teeth.

Therefore, the aim of the present study was mainly to distinguish the different periodontal statuses of teeth opposing dental implants and 3-unit FPDs through identification of the cytokine profile in the GCF. In addition, based on the above relationship, we preliminarily evaluated whether cytokine levels in GCF changed due to increased occlusion force caused by poor osseoperception of dental implants, thus highlighting the need to clinically observe contact teeth in patients with dental implants.

## Material and methods

### Ethical considerations

The present study was approved by the Clinical Research Ethics Committee of the Beijing Haidian Hospital (Grant No. 2019024) and conducted in accordance with the 2013 revised version of the Declaration of Helsinki. All participants were required to sign a consent form before participating in the study.

### Exclusion criteria

Individuals were excluded if they were older than 65 years; had missing teeth without restoration; were previously diagnosed with a systemic disease; had periodontal disease or used any medication affecting the periodontal parameters; had received periodontal, antibiotic or occlusal adjustment treatment within the three months prior to the study or were under orthodontic treatment prior to the study; presented necrotizing ulcerative gingivitis; were smokers or had alcohol dependence; had unstable occlusal relationships or had parafunctional habits, such as clenching and bruxism; or refused to sign the consent form.

### Inclusion criteria

Only the first molar was missing unilaterally in the mandibular posterior teeth area. There are no missing teeth in the posterior tooth area of missing teeth. Stable occlusion can be obtained after tooth restoration. The restoration method of missing teeth is implant or three-unit fixed bridge with natural teeth on both sides of missing teeth as abutment.

### Study design and patient groups

A total of 162 individuals with a medical record of dental implants or 3-unit FPDs restoring one side of the lower first molar visited the Department of Stomatology of Beijing Haidian Hospital during the period from April 2017 to August 2020. Since 88 patients met the exclusion criteria, 74 participants were recruited into this cross-sectional study.

### Sociodemographic and dental characteristics

Basic sociodemographic characteristics (age, sex, and BMI), frequency of dental hygiene equipment use (toothbrushing and dental floss), smoking habit, and most recent visit to a dental hygienist or dentist were recorded.

### Clinical evaluation

The clinical examination was evaluated by a single qualified investigator (Dr. Zhu).

For the periodontal examination, the periodontal probing depth (PPD), bleeding on probing (BOP), plaque index (PI) and clinical attachment loss (CAL) were recorded for the whole mouth of participants. Periodontal health was defined as no or minimal levels of clinical inflammation in a periodontium with normal support (clinical periodontal health: no/minimal bleeding on probing [BOP < 10%], normal gingival sulcus depth and bone height, controlled modifying and predisposing factors) [13].

For the occlusal force examination, the T-Scan III system (Tekscan, USA) was used following a procedure in which patients were instructed to clench their teeth in the maximal intercuspal position before they could perform the clenching movement correctly. After that, the patients were told to sit in a relaxed upright position in the dental chair and then were instructed to clench their teeth firmly on the sensor 3 times [2]. The expressed relative occlusal force (ROF) of the selective upper first molar region (ROF:U6), the selective posterior region (ROF:U4-7), and the selective side of upper dentition (ROF:U1-7), as the percentage of the overall ROF, was recorded.

### Gingival crevicular fluid (GCF) samples

After the gingival plaque was removed and the target sites were dried, six sterile Whatman 3 MM papers were gently inserted into the gingival crevice of the selective molars (mesiobuccal, midbuccal, distobuccal, mesiopalatal/lingual, midpalatal/lingual, and distopalatal/lingual) for 30 s. Based on previous research and laboratory examination, this standardized collection period ensured that the cytokine detection process was performed according to the manufacturer's instructions [14, 15]. The samples were further transferred to sterile 1.5 mL tubes separately and stored at − 80 °C until use [16].

### Cytokine detection

The GCF collected on the papers was diluted with 500 µL of sample diluent provided with the Human Cytokine Antibody Array Kit (QAH-PDD-1, RayBiotech, USA). The protein concentrations of all the samples were measured by the BCA method. To detect the specific cytokine concentrations, all of the samples were diluted again to a concentration of 300 μg/ml. Then, a total of 60 µL of each sample was added to the array. Twenty cytokines, including C-reactive protein (CRP), interferon-γ (IFN-γ), IL-1, IL-2, IL-4, IL-6, IL-8, IL-10, IL-12, IL-17, matrix metalloproteinase (MMP)-1, MMP-9, MMP-13, osteoprotegerin (OPG), osteopontin (OPN), osteoactivin (OCN), receptor activator of NF-κB (RANK), transforming growth factor-β1 (TGF-β1), and TNF-α, were detected according to the manufacturer’s instructions.

Briefly, the arrays were incubated with GCF samples for 2 h at room temperature (RT) after blocking. Then, the arrays were washed with PBS three times and incubated with biotin-conjugated antibodies for 2 h at RT. Furthermore, the arrays were incubated with Cy3 equivalent dye-conjugated streptavidin in the dark for 1 h at room temperature after being washed with PBS. Finally, the arrays were visualized with a laser scanner, and the data were analyzed with Axon GenePix software [14].

### Statistical analysis

The Mann–Whitney test was used to study the association of cytokines and dental implants and sex and the sociodemographic, periodontal and occlusal parameters between dental implants and 3-unit FPD patients. Chi-square tests were used for categorical variables. Spearman's correlation coefficient was used to study the correlation of cytokines with age, BMI and ROF:U6. The Kruskal–Wallis test with Bonferroni correction was used to analyze the association of cytokines with years after final restoration. We performed binary logistic regression analyses with the outcome of dental implant patients using age, BMI, ROF:U6, and the cytokine in question as predictive factors of the two groups. A *p* value less than or equal to 0.05 determined using SPSS 23.0 (IBM, USA) was considered statistically significant.

## Results

### Clinical findings

Table 1 shows the sociodemographic, periodontal and occlusal parameters of the participants. Thirty-one out of the 74 study participants were assigned to the 3-unit FPD group, and the other 43 participants were assigned to the dental implant group. There were no differences in the sociodemographic characteristics between the two groups. The frequency of dental hygiene equipment use was evenly distributed between the two groups. All of the patients had regular dental health care at least once a year or more often. Three-unit FPD patients had longer years after the last visit in the clinic. There was no difference in regular periodontal examination values. However, the ROF for the selective first molar region measured by T-scan was significantly higher in the dental implant group. There was no difference in the ROF in adjacent posterior teeth or hemiarch between the two groups.

**Table 1:**
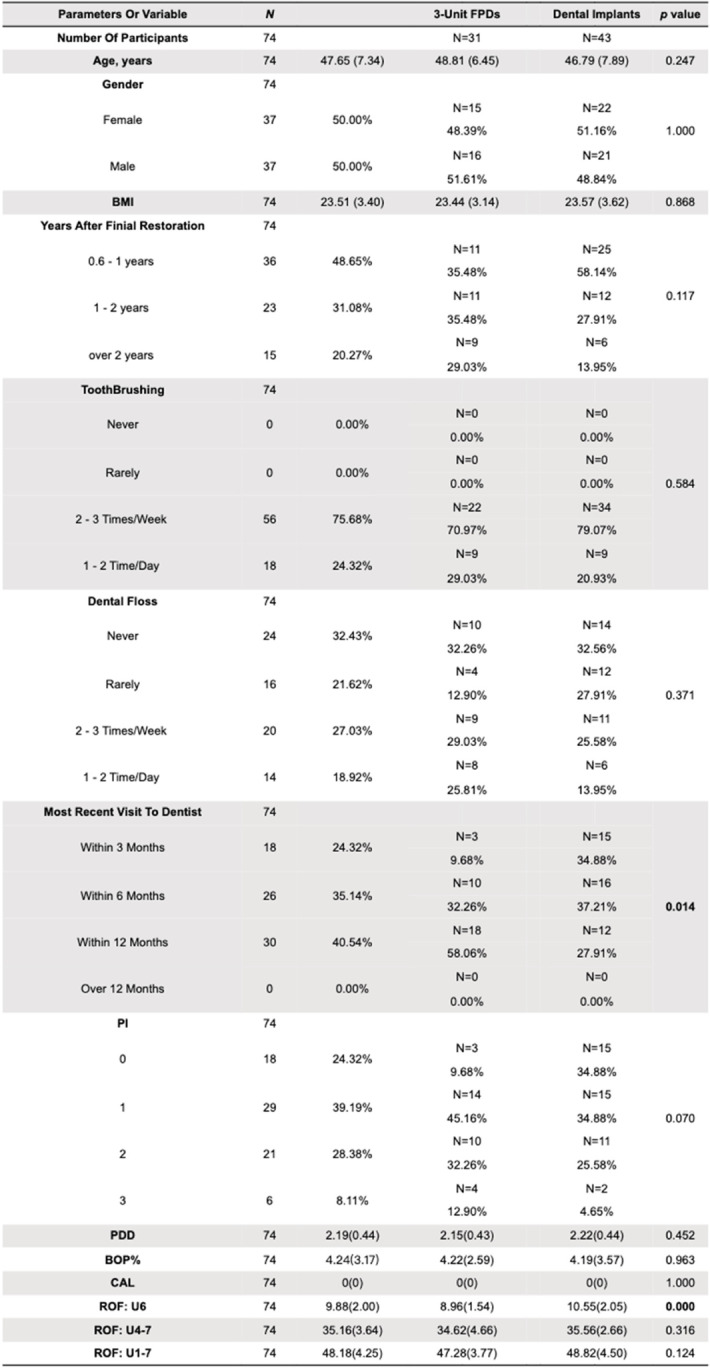
Stratified sociodemographic, periodontal and occlusal parameters for dental implants and 3-unit FPDs

### GCF content of biological mediators

Table 2 shows the levels (pg/ml) of the pro-inflammatory cytokines and anti-inflammatory cytokines in the two groups measured by the commercial chip. According to the manufacturer’s instructions, 150 pg/ml was set as the LOD value for the chip to eliminate the influence of background interference. Thus, all the cytokines were successfully detected in the teach sample.

**Table 2:**
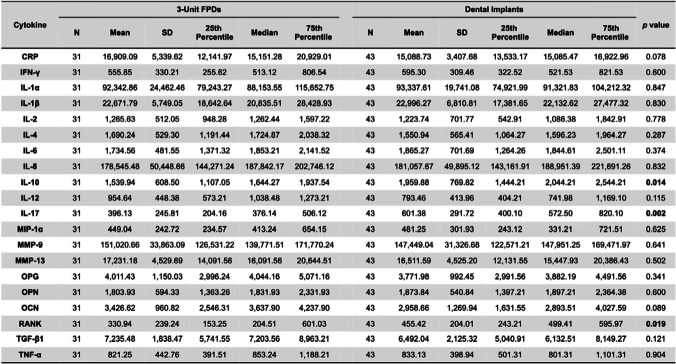
Cytokine levels in GCF stratified by dental implants and 3-unit FPDs

**Fig. 1:**
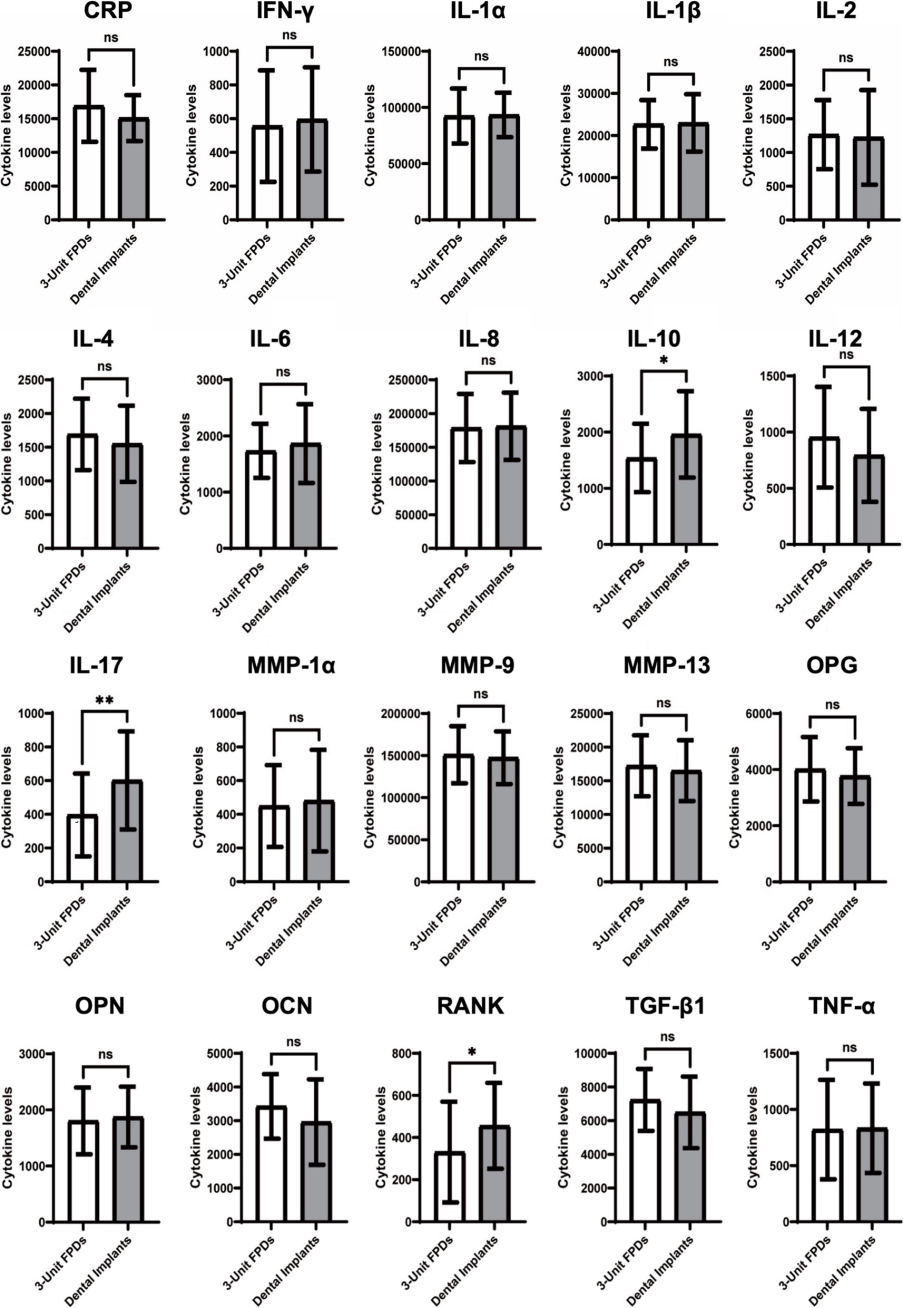
Different cytokine expression levels in GCF of 3 units of FPDs and dental implants

Figure 1 shows that the levels of IL-10, IL-17 and RANK in the stratified dental implant group were significantly higher than those in the 3-unit FPD group. No differences were found between those two groups for the other 18 cytokine expression levels.

### Cytokines stratified for sociodemographic, periodontal and occlusal parameters

Table 3 shows all twenty cytokines stratified for age, BMI, sex, ROF: U6, ROF: U4-7, ROF: U1-7, PPD, BOP, sex, years after final restoration, tooth brushing, dental floss, most recent visit to dentist, and PI. There was an association between IFNg, IL-1α and BMI. IL-10, IL-17 and RANK were associated with ROF:U6. IL-10, IL-17 and POS1 were associated with ROF:U4-7. IL-2, IL-12 and MMP-1α were associated with PPD. POS2 and TGF-β1 were associated with BOP. IL-6 and OPN were associated with sex, and IL-1α was associated with years after final restoration. OPG was associated with dental floss. IL-1β was associated with the most recent visit to a dentist. IL-1α and IL-8 were associated with PI.

**Table 3:**
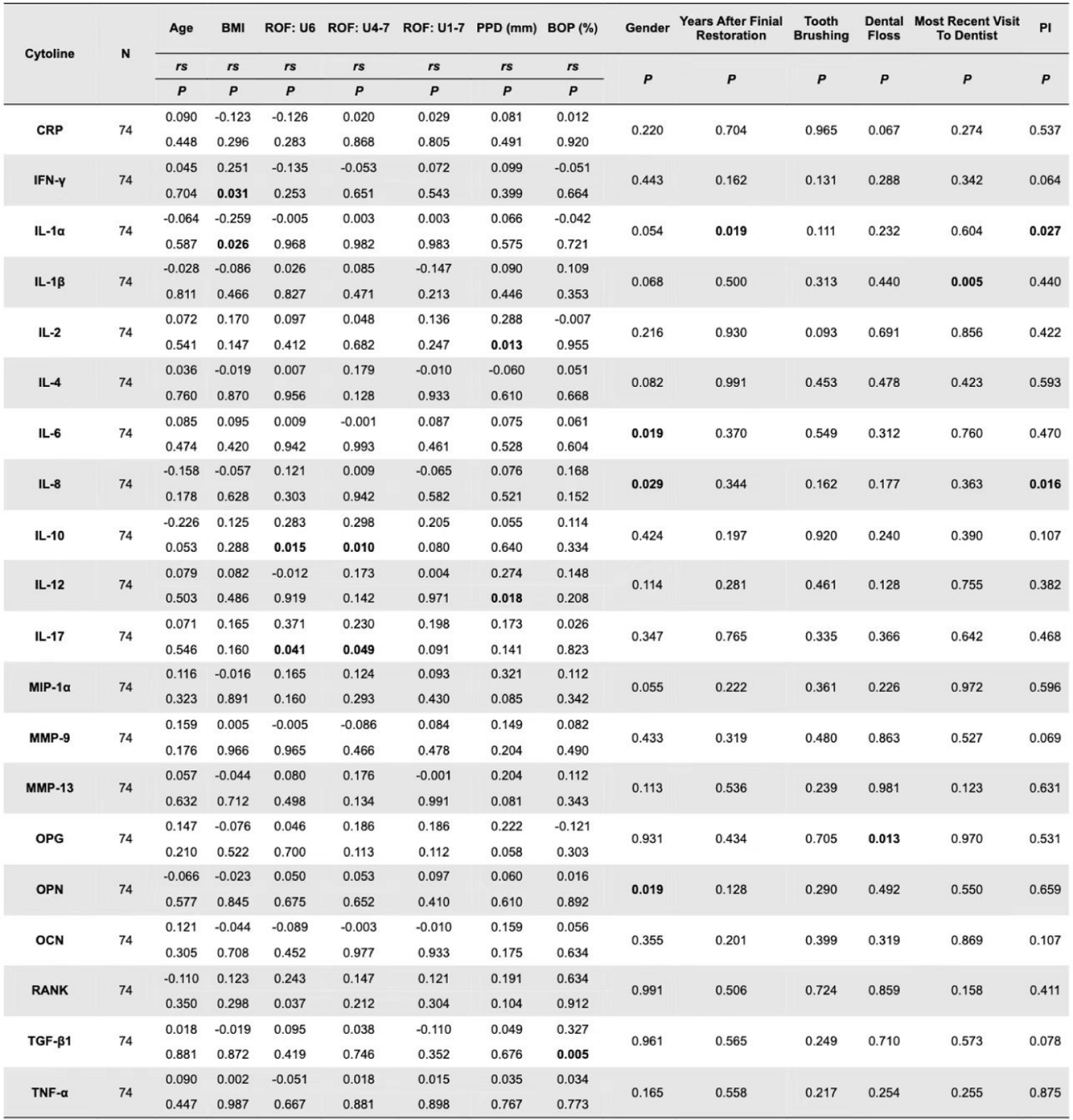
Cytokine levels stratified by sociodemographic, periodontal and occlusal parameters

### Binary logistic regression analyses

The levels of IL-10, IL-17 and RANK were found to be different in the dental implant group and the 3-unit FPD group. We used binary logistic regression to further analyze the association of the above cytokines and the dental implant group. We only found a weak association between IL-10 and the dental implant group (IL-10 *p* = 0.021, OR 1.001, 95% CI 1.000–1.002). No association was found between IL-17 and dental implants (*p*= 0.147) or RANK and dental implants (*p*= 0.291).

## Discussion

This cross-sectional study aimed to evaluate the cytokine expression level around teeth opposing dental implants and 3-unit FPDs. IL-10, IL-17 and RANKL were significantly higher in the dental implant group. There was only a weak association between IL-10 and dental implants. IL-10 was associated with ROF:U6, which indicated the effect of poor osseoperception of dental implants.

The occlusal loading between natural teeth and between dental implants and natural teeth exhibits many differences. A suitable occlusal force could stimulate the development of the cranio-maxillofacial system and cause physiological wear of the teeth. However, the abnormal contact of the teeth caused by dysfunction of the masticatory system, malocclusion and bruxism could cause excessive occlusal forces and lead to pathological occlusal loading. Previous research has shown the link between occlusion trauma and periodontal disease [17]. Therefore, this study excluded those patients and mainly focused on the differences between dental implants and natural teeth. In addition, to minimize the variation within the implant groups, we used 10 mm-long × 4.8 mm-diameter titanium screw-type bone-level implants from Straumann, and to minimize the variation within two groups, the same zirconia all-ceramic restorations were used. In this study, we still found an increased occlusal force for teeth opposing dental implants compared to those in contact with 3-unit FPDs. Considering that the sociodemographic characteristics and clinical parameters except the most recent visit to dentist were comparable in all the participants, the differential expression pattern of the cytokines in the GCF might reflect the poor osseoperception of dental implants. The shorter years for the dental implant group in this study are probably because the patients with dental implants need a longer treatment period. Considered clinicians currently tend to set a light occlusion after implant-supported dental restorations are placed, which might lead to the continuous eruption of the opposing teeth. Previous research has shown that these changes occurred within the first 6 months after restoration insertion [18]. We found an association between IL-1α and years after final restoration. However, as a cross-sectional study, it did not allow us to assess whether the study individuals had stable cytokine expression conditions.

Dysregulated production of cytokines could be seen as a predictor of various infectious and inflammatory diseases. In our study, although little difference was detected for the clinical parameters, teeth opposing implants showed higher cytokine levels in GCF than those opposing 3-unit FPDs for IL-10, IL-17, and RANK, including both proinflammatory cytokines and anti-inflammatory cytokines. Previous research has shown that IL-10 can negatively regulate IL-17 within innate immune cell populations by mitigating chemokine CXCL5 and CXCL1 expression, which inhibits inflammation and bone loss in periodontal tissue [19]. In addition, RANK is involved in regulating bone remodeling via the OPG/RANKL signaling pathway [20]. Taken together, the higher expression cytokine levels in the dental implant group indicated higher tissue remodeling activity, which was consistent with previous research showing that several inflammation-related cytokine levels around healthy implants were higher than those around natural teeth since dental implants and natural teeth have different anatomic, vessel and nerve environments, core microbiomes and immune characteristics [21, 22].

Previous research has demonstrated that several factors, such as periodontal inflammation, tissue damage, and occlusion trauma, change cytokine expression levels in GCF [23–25]. In addition, those variations could occur even in different sites of a single tooth. Recent research has shown different cytokine levels between peri-implant sites and periodontitis sites since dental implants and natural teeth have different plaque compositions [26, 27]. To the best of our knowledge, little is known about the cytokine expression pattern around teeth opposing dental implants or 3-unit FPDs. Compared to occlusion loading, general factors such as materials for dental implants and 3-unit FPDs and bacterial composition are unlikely to influence opposing teeth. In addition, most of the variation in this cross-section study was evenly distributed in the two groups, except for the occlusion forces measured by T-scan. Therefore, the cytokines that varied in the two groups were likely ascribed to the occlusion factor. The preliminary outcomes in this study also showed that IL-10 is associated with the dental implant group, and IL-10 is also associated with occlusal forces in the first molar region, which further confirms that dental implants could affect opposing tooth cytokine expression levels by increasing site occlusion loading. Previous research has also noticed that occlusal stress triggers increases in the proinflammatory cytokines IL-1β, IL-6 and IL-17. In addition, another study showed that implants with a higher occlusal load presented higher levels of IL-10 in peri-implant crevicular fluid, which indicated the role of IL-10 in monitoring the occlusal force and counterbalancing the increased proinflammatory cytokines [28].

Overall, opposing dental implants could increase the levels of cytokines in the GCF compared to the 3-unit FPD patients. Since the ROF was weakly associated with dental implant patients and previous research has shown that higher occlusal forces could lead to bone resorption [29, 30], changes in the occlusal force of teeth opposing implants or natural teeth might play an important role in modulating the cytokine expression pattern. However, the current study has several limitations. First, the implant restoration is composed of a stent and a fixed restoration, the 3-unit fixed bridge is composed of two stents and a component, and there is a difference in pressure between their compensation types. However, since the main purpose of this study is to explore the effects of the two most common methods of restoring a single missing tooth on the periodontal state of the opposing teeth, it has certain reference value for the selection of clinical treatment options. At the same time, the transmission of occlusal force to the opposing teeth caused changes in cytokines in the gingival crevicular fluid, which minimized the above differences. Second, because the bite force is constantly changing after dental implantation, it is necessary to study the relationship between the bite force change and cytokine level longitudinally. Finally, the expression of cytokines in the implant group was significantly higher than that in the 3-unit FPD group. Therefore, further research is needed to prove whether this process is involved in regulating the periodontal tissue of teeth. As far as we know, due to ethical issues, most of the scientific literature on the effects of occlusal load on periodontal tissue health has focused on animal studies [31]. The present study provides preliminary information on different expression levels of cytokines present in teeth opposing dental implants and 3-unit FPDs.

## Conclusions

This study demonstrated that teeth opposing implants have a higher level of cytokines in the GCF than teeth opposing 3-unit FPDs in periodontally healthy participants because of the poor osseoperception of dental implants. IL-10 might reflect a higher occlusion force in the dental implant region.

## Data Availability

All data generated or analyzed during this study are included in this published article.
